# Molecular dynamic (MD) studies on Gln233Arg (rs1137101) polymorphism of leptin receptor gene and associated variations in the anthropometric and metabolic profiles of Saudi women

**DOI:** 10.1371/journal.pone.0211381

**Published:** 2019-02-14

**Authors:** Maha Daghestani, Rituraj Purohit, Mazin Daghestani, Mamoon Daghistani, Arjumand Warsy

**Affiliations:** 1 Department of Zoology, Director of Central Laboratory, Female Center for Scientific & Medical Colleges, King Saud University, Riyadh, Saudi Arabia; 2 Biotechnology Division, CSIR-Institute of Himalayan Bioresource Technology (Council of Scientific & Industrial Research), Palampur (H.P.) India; 3 Department of Obstetrics and Gynecology, Umm Al-Qura University, Makkah, Saudi Arabia; 4 Department of Surgery, King Abdulaziz Medical City, National Guard Health Affairs, Jeddah, Saudi Arabia; 5 Central Laboratory, Female Center for Scientific & Medical Colleges, King Saud University, Riyadh, Saudi Arabia; Dasman Diabetes Institute, KUWAIT

## Abstract

The Gln233Arg (A>G; rs1137101) polymorphism of the leptin receptor gene (LEPR) has been investigated extensively and is reported to be associated with different metabolic states. In this investigation, we aimed to study the frequency of Gln233Arg genotypes and alleles in a group of Saudi women stratified by their body mass index (BMI), to correlate the LEPR genotypes with variations in anthropometric, lipid and hormonal parameters and to investigate conformational and structural variations in the mutant LEPR using molecular dynamic (MD) investigations. The study group included 122 Saudi women (normal weight = 60; obese = 62) attending the clinics for a routine checkup. Anthropometric data: height, weight, waist and hip circumference were recorded and fasting serum sample was used to estimate glucose, lipids, ghrelin, leptin and insulin. BMI, W/H ratio, and HOMA-IR values were calculated. Whole blood sample was used to extract DNA; exon 6 of the LEPR gene was amplified by PCR and sequencing was conducted on an ABI 3100 Avant Genetic Analyser. Molecular Dynamic Simulation studies were carried out using different softwares. The results showed the presence of all three genotypes of Gln233Arg in Saudi women, but the frequencies were significantly different when compared to reports from some populations. No differences were seen in the genotype and allele frequencies between the normal weight and obese women. Stratification by the genotypes showed significantly higher BMI, waist and hip circumference, leptin, insulin, fasting glucose and HOMA-IR and lower ghrelin levels in obese women carrying the GG genotype. Even in the normal weight group, individuals with GG genotype had higher BMI, waist and hip circumference and significantly lower ghrelin levels. The MD studies showed a significant effect of the Gln/Arg substitution on the conformation, flexibility, root-mean-square fluctuation (RMSF), radius of gyration (Rg) values, solvent-accessible surface area (SASA) and number of inter- and intra-molecular H-bonds. The results suggest that the structural changes brought about by the mutation, influence the signaling pathways by some unknown mechanism, which may be contributing to the abnormalities seen in the individuals carrying the G allele of rs1137101.

## Introduction

The adipose-specific hormone leptin (LEP; 164160), regulates energy expenditure, satiety, and adipose tissue mass through hypothalamic effect, by binding to the leptin receptor (LEPR, OB-R; CD295), which is a single transmembrane–domain receptor belonging to the cytochrome family receptors [1–6]. It also plays essential roles by stimulating immune and barrier cells and hence promotes the activation, proliferation, cell death resistance and wound repair [7]. It exerts its action on the target cells by binding with high affinity, to the cytokine receptor homology-2 (CRH2) domain, of the LEPR. This binding induces clustering of the activated receptor complexes, Janus Kinase 2 (JAK2), which catalyzes the phosphorylation of intracellular tyrosine residues. The phosphorylation leads to the recruitment of several transcription factors, one of which is Signal Transducer and Activator of Transcription 3 (STAT3). The STAT3 becomes activated and dimerizes, and is translocated to the nuclear region, where it initiates a transcriptional program for several genes [8–10].

The hormone, leptin, seems to be involved in the pathophysiology of several diseases and metabolic states, including obesity and diabetes mellitus type 2 [11,12]. Its receptor, LEPR, is present in the cell membranes of a wide variety of cells of different tissues, signifying the essential physiological role played by leptin [13–17]. The LEPR is a protein of 1,165 amino acids grouped in several domains. There are two extracellular domains CRH1 and 2, where CRH1 is essential for the high-affinity interaction, and the CRH2 domain is required for leptin binding [18]. The LEPR is encoded by the LEPR gene, located at 1p31.3 and has 24 exons. Several mutations and single nucleotide polymorphisms have been reported, both in the coding and non-coding regions of this gene [4, 19, 20]. Several of these variations influence the LEPR functions and result in reduced functioning of the LEPR. In some of the variants, the leptin cannot bind to the receptor, while in others the leptin binds, but there is either no or low response to this binding. The resultant leptin deficiency state is associated with a variety of abnormal states. Several genetic variations in the LEPR gene are reported that alter the normal functional capacity of the LEPR protein, resulting in an apparent “leptin receptor deficiency”, hence preventing either the binding of leptin to the receptor or the receptor from responding to the bond leptin. The consequences of these abnormalities are excessive hunger, weight gain, and hypogonadotropic hypogonadism, where the affected individuals experience extreme obesity, hyperphagia, an absence of or delay in puberty and later may develop infertility disorder [19, 20].

A mutation in the exon 6 of LEPR ‘rs1137101 (Gln223Arg)’, has been extensively investigated. It is frequently reported and exhibits polymorphism in several populations [20]. It is an A to G transition, where a codon CAG changes to CGG, resulting in the substitution of glutamine by arginine (Gln223Arg) in the LEPR. The frequencies reported by the 1000 genomes project, for the A allele are 41%, and for the G allele are 59% [21], but wide variations in the frequencies are reported in different populations and ethnic groups [22].

Stratigopoulos and coworkers showed that the Gln223Arg polymorphism existed in the N-terminal CRH1 domain and studied its functional consequences [21]. Several reports linked it to some abnormalities, such as obesity and adiposity [23–26], type 2 diabetes mellitus [27], peritonitis [28], susceptibility to enteric and respiratory disorders [29–33], arteriosclerosis [34] and multiple forms of cancer [35–37]. However, other studies failed to show any association [38–40].

In an elegant study, Verkerke and coworkers [41] determined whether the Gln223Arg amino acid replacement in LEPR has an effect on the binding kinetics of the leptin to the receptor, by measuring the leptin/LepR interaction using SPR technology on a BiaCore T200 platform. They showed that the Gln223Arg, located in the extracellular domain of LEPR, does not affect the rate at which leptin binds to and dissociates from its receptor. They suggested possible post-receptor defects as a cause of the abnormalities that result from the presence of the mutant LEPR.

We designed this study to conduct MD Simulation studies on native and mutant LEPR, in an attempt to determine the changes taking place in the variant at the atomic level. We also determined the genotype frequency of the three genotypes of the Gln233Arg polymorphism and correlated the anthropometric, biochemical and hormonal parameters to the different genotypes in normal weight and obese Saudi women.

## Materials and methods

The local Ethics Committee at the Umm Al Qura University, Makkah Al Mukaramah, Saudi Arabia, approved this study (IRB No. 235). The study was conducted on 124 Saudi women (normal weight and obese) attending the Clinics at health centers in Makkah, Saudi Arabia, for a routine check-up. Informed consent was obtained from each female who volunteered to take part in the study and signed an informed consent.

Exclusion criteria for all the subjects included congenital adrenal hyperplasia, Cushing’s syndrome, hyperprolactinemia, hypothyroidism, current or previous (within the last six months) use of oral contraceptives, anti-androgens, ovulation induction agents, glucocorticoids, anti-diabetic and anti-obesity drugs or other hormonal medicines. None of the normal weight women had metabolic, cardiovascular disorder, neoplastic or other concurrent chronic illness such as hepatic disorders, renal disease, and diabetes. All the subjects were non-smokers and had normal physical activity.

### Anthropometric measurements

For the study group weight and height were recorded and BMI (Kg/m^2^) was calculated; waist and hip circumference were measured in the standing position, and waist-hip ratio (WHR) was calculated.

### Biochemical measurements

The women were asked to visit the clinic following a 12 hour fasting state and fasting blood sample (5ml) was drawn in plain red-top tubes for the determination of lipids, leptin and insulin in the serum. Ethylene diamine tetra acetate (EDTA) tubes and aprotinin (500 KIU/ml; Trasylol; Bayer Corp., Leverkusen, Germany) were used to collect 2 ml blood for DNA extraction and estimation of total ghrelin, respectively. Blood was drawn in fluoride tubes for glucose estimation.

Enzyme immunoassay kit (EIA) from Phoenix Pharmaceuticals, Inc., (Belmont, CA, USA) was used for determination of total ghrelin levels. Insulin levels were estimated in serum using the electro-chemiluminescence immunoassay ‘‘ECLlA” on a Roche Elecsys 1010/2010 and MODULAR ANALYTICS E170 (Elecsys module) immunoassay analyzers (Roche Diagnostic, Mannheim, Germany). Plasma glucose was estimated using the glucose oxidase method on a Beckman Glucose Analyzer (Fullerton, CA). Total serum cholesterol, triglycerides, high-density lipoprotein [HDL-cholesterol] and low-density lipoprotein [LDL-cholesterol] were determined by enzymatic methods using commercial kits (Boehringer Mannheim). Leptin concentrations were determined by ELISA Kit (Phoenix Pharmaceuticals).

The homeostatic model assessment insulin resistance (HOMA-IR) was calculated using the following formulas:

HOMA‑IR=Fastingseruminsulin(μU/ml)×Fastingplasmaglucose(mmoll‑1)/22.5)

### Genotyping of ‘rs1137101’ polymorphism in exon 6 of LEPR gene

The genomic DNA of all subjects was extracted from peripheral blood leukocytes using Gentra Systems Kit (Minneapolis, MN, cat # D5500). The DNA fragment containing codons of the exon 6 of LEPR gene was amplified by polymerase chain reaction (PCR) using a sense primer (5'- TATAGGCCTGAAGTGTTAGAAG-3') and an antisense primer (5'- CCCATATTTATGGGCTGAACT -3'). The PCR conditions were as follows: initial denaturation step at 95°C for 15 minutes, 34 cycles of denaturation at 95°C for 1 minute, annealing at 55°C for 1 minute, and extension at 72°C for 1 minute, with a final extension of 10 minutes at 72°C. With these primers, a PCR product was obtained and was visualized using ethidium bromide, following electrophoresis on agarose gel electrophoresis, Nucleotide sequencing was carried out by the ABI Big Dye Terminator protocol using ABI 3100 Avant Genetic Analyzer.

### Homology modelling

The 3-D structure of the LEPR protein was not available in Protein Database (PDB). The amino acid sequence (UniProt id P48357, length from 22 to 1165) of native and mutant (Gln223Arg) LEPR were submitted to LOMETS metaserver [42] for homology modeling. The server generated multiple models of which those produced by HHSEARCH and SP3 algorithms using appropriate templates respectively, were considered for further analysis. The accuracy and quality of homology model was verified employing the PROCHECK [43] and PROSA [44] programs and Ramachandran plot using the RAMPAGE server (http://mordred.bioc.cam.ac.uk/~rapper/rampage.php).

### Molecular dynamics simulation

Model structure of native and mutant leptin protein was used as starting point for MD simulations. We used similar simulation protocols which were used in our previous studies [44–48]. In this simulation we implemented 20 ns for position restraint to allow solvent molecules to enter the cavity region of structure and MD simulation (production run) for 120 ns. We then computed the comparative analysis of structural deviations in native and mutant structure. Root-mean-square fluctuation (RMSF), radius of gyration (Rg) and solvent accessible surface area (SASA) analysis were carried out by using gmx rmsf, gmx gyrate and gmx sasa tools, respectively. Number of distinct hydrogen bonds formed by specific residues to other amino acids within the protein during the simulation (h-bonds) was calculated using g_hbond. NH bond were determined on the basis of donor–acceptor distance smaller than 0.35 nm and of donor-hydrogen-acceptor. All the graphs were plotted using XMGRACE [49].

### Statistical analysis

Data obtained for the study groups were entered into Excel spreadsheets and analyzed using Statistical Package for the Social Science, version 22, (SPSS; Inc., Chicago, IL, USA). The mean ± SEM were obtained for each parameter. Comparison of the results between any two groups was carried out using Student’s t-test or Mann-Whitney U-test as appropriate.

The genotype was established for each sample by comparing with the reference sequence (Genebank) using DNAstar program. Genotype and allele frequencies for normal weight and obese group were calculated manually. For comparison of genotype and allele frequencies between the two groups (obese and normal weight), Odds Ratio (OR), 95% Confidence Interval (CI), Chi-square (χ^2^) and p-value were obtained using https://ihg.gsf.de/cgi-bin/hw/hwa1.pl. For all comparisons a p-value <0.05 was considered statistically significant.

## Results

The study group included 122 women of Saudi origin [normal weight: BMI ≤25 = 60; obese: BMI >30 kg/m^2^ = 62).

The anthropometric, metabolic and hormonal parameters of the study groups are presented in Table 1. The genotype frequencies for the three genotypes (AA, AG and GG) in the study group were calculated, allele frequencies were obtained and compared, and the results are presented in Table 2. The major allele ‘A’ and minor allele 'G" occur at frequencies of 79.84% and 20.16%, respectively in the normal weight Saudis.

**Table 1:** The anthropometric measurements and metabolic profile of the normal weight and obese group.

Parameter	Normal weight (Mean±SEM)	Obese Group (Mean±SEM)	p
BMI (kgm2)	20.85±0.25	34.47±0.73	0.0001
Waist (cm)	66.85±0.69	95.16±1.94	0.0001
Hip (cm)	94.59±0.91	117.38±1.88	0.0001
WH ratio	0.70±0.005	0.81±0.007	0.0001
Cholesterol (mmolL)	3.42±.057	3.86±0.072	0.0001
Triglyceride (mmolL)	0.68±0.03	1.04±0.06	0.0001
HDL (mmolL)	1.44±0.040	1.11±0.038	0.0001
LDL (mmolL)	1.31±0.048	2.11±0.078	0.0001
Leptin nglml	11.70±0.46	39.94±2.59	0.0001
Fast ghrelin nglml	0.57±0.015	0.33±0.014	0.0001
Fasting Insulin (pmolL)	52.57±2.28	93.51±5.37	0.0001
Fasting Glucose	4.53±0.05	4.89±.065	0.0001
HOMAR-IR	0.96±.041	1.72± .098	0.0001

SEM = Standard error of the mean

Normal cut off for lipids

Cholesterol = <5.7 mmol/L; Triglycerides = 0.45–1.71 mmol/L; LDL-cholesterol = <3.4 mmol/L

HDL-cholesterol = >0.91 [mmol/L]

**Table 2:** Comparison of the genotype and allele frequencies of Gln233Arg genotypes in the obese and lean groups.

Variation	Normal weight group No. (%)	Obese group No. (%)	OR	CI	χ^2^	p-value
AA	42 (67.7)	39 (62.9)	0.37	0.122–1.15	3.08	0.079
AG	15 (24.20)	10(16.13)	0.76	0.11–1.03	3.82	0.049
GG	5 (8.04)	13 (20.96)	2.66	0.869–8.12	3.08	0.079
Allele frequencies						
A	99 (79.84)	88 (70.97)	0.64	0.36–1.12	2.19	0.139
G	25 (20.16)	36 (29.03)	1.55	0.86–2.8		

The study group was separated into the obese and normal weight groups and the genotypes and allele frequencies of Gln233Arg were separately calculated and compared. The results are presented in Table 2. The G allele occurred at a higher frequency in the obese women, but the difference compared to the normal weight group was not statistically significant. The genotype frequencies were also different, but the difference was significant only in the frequencies of AA vs AG genotypes (p = 0.049).

The expected genotype was calculated and the Hardy-Weinburg Equilibrium was applied. The Hardy-Weinburg equilibrium was obeyed by the normal weight group (p<0.05), but was disturbed in the obese population (p<0.05)

In an attempt to study the effect of the different LEPR genotypes on the anthropometric, biochemical and hormonal parameters in the normal weight and obese women, we analyzed the data based on the LEPR genotypes: AA, AG and GG. The results in the three genotypes in the normal weight and obese groups were compared using Students‘t’ test, and the significance of the difference in the results were recorded. The results are presented in Table 3.

**Table 3:** Value of anthropometric, biochemical and hormonal parameters in the normal weight and obese women grouped according to their Gln233Arg genotypes (AA, AG, GG].

Parameter	Normal weight group			P value	Obese group			P value
	LEPR Genotype				LEPR Genotype			
	AA	AG	GG		AA	AG	GG	
BMI (kgm2)	20.37±0.26	21.51±0.5	23.17±0.8	0.001^a^ 0.040^b^ 0.100^c^	32.08±.8	39.77±2.20	38.01±1.70	0.0001 ^a^ 0.0001 ^b^ 0.529 ^c^
Waist (cm)	65.95±0.78	67.61±.78	72.40±1.7	0.009^a^ 0.324 ^b^ 0.070 ^c^	89.03±1.75	108.90±5.5	104.17±4.27	0.0001 ^a^ 0.006 ^b^ 0.492 ^c^
Hip (cm)	93.59±1.08	95.4±1.84	101.0±2.4	0.028 ^a^ 0.422 ^b^ 0.095 ^c^	110.3±1.7	132.20±4.0	128.50±3.46	0.0001 ^a^ 0.0001 ^b^ . 0.492 ^c^
WH ratio	0.70±0.007	0.71±0.01	0.72±0.02	0.462 ^a^ 0.841 ^b^ 0.466 ^c^	0.81±0.01	0.82±0.02	0.81±.02	0.870 ^a^ 0.481 ^b^ 0.702 ^c^
Cholesterol (mmolL)	3.38±0.07	3.50±0.12	3.52±0.20	0.511 ^a^ 0.409 ^b^ 0.935 ^c^	3.79±0.09	4.17±0.14	3.82±.14	0.881 ^a^ 0.036 ^b^ 0.095 ^c^
Triglyceride (mmolL)	0.67±0.04	0.71±0.06	0.71±0.08	0.755 ^a^ 0.662 ^b^ 0.975 ^c^	0.99±.07	1.20±0.16	1.07±.11	0.586 ^a^ 0.213 ^b^ 0.496 ^c^
HDL (mmolL)	1.45±0.049	1.42±0.09	1.41±0.10	0.827 ^a^ 0.842 ^b^ 0.946 ^c^	1.14±.05	1.15±0.11	1.01±.05	0.079 ^a^ 0.168 ^b^ 0.229 ^c^
LDL (mmolL)	1.31±0.06	1.33±0.09	1.18±0.07	0.460 ^a^ 0.898 ^b^ 0.233 ^c^	2.08±.09	2.44±0.21	1.90±.16	0.329 ^a^ 0.111 ^b^ 0.050 ^c^
Leptin nglml	11.32±0.47	12.50±1.48	12.80±1.02	0.302 ^a^ 0.460 ^b^ 0.870 ^c^	33.68±2.6	51.20±6.54	51.42±7.02	0.005 ^a^ 0.006 ^b^ 0.982 ^c^
Fast ghrelin nglml	0.60±0.019	0.52±0.02	0.44±0.02	0.001 ^a^ 0.008 ^b^ 0.045 ^c^	0.379±.01	0.226±0.02	0.233±.02	0.0001 ^a^ 0.0001 ^b^ 0.791 ^c^
Fasting Insulin (pmolL)	51.61±2.81	58.4±4.85	45.4±4.84	0.046 ^a^ 0.240 ^b^ 0.082 ^c^	82.56±6.9	109.96±9.7	116.30±9.54	0.009 ^a^ 0.033 ^b^ 0.649 ^c^
Fasting Glucose	4.52±0.06	4.5±0.12	4.62±0.21	0.580 ^a^ 0.240 ^b^ 0.680 ^c^	4.82±.07	4.80±0.17	5.26±0.13	0.008 ^a^ 0.932 ^b^ 0.039 ^c^
HOMA-IR	0.84±.09	1.06±.08	0.95±0.05	0.480 ^a^ 0.160 ^b^ 0.250^c^	1.52±.13	2.01±0.18	2.16±0.17	0.007 ^a^ 0.040 ^b^ 0.566^c^

a- AA vs GG

b- AA vs AG

c- GG vs AG

### Results of MD studies

**Fig 1:**
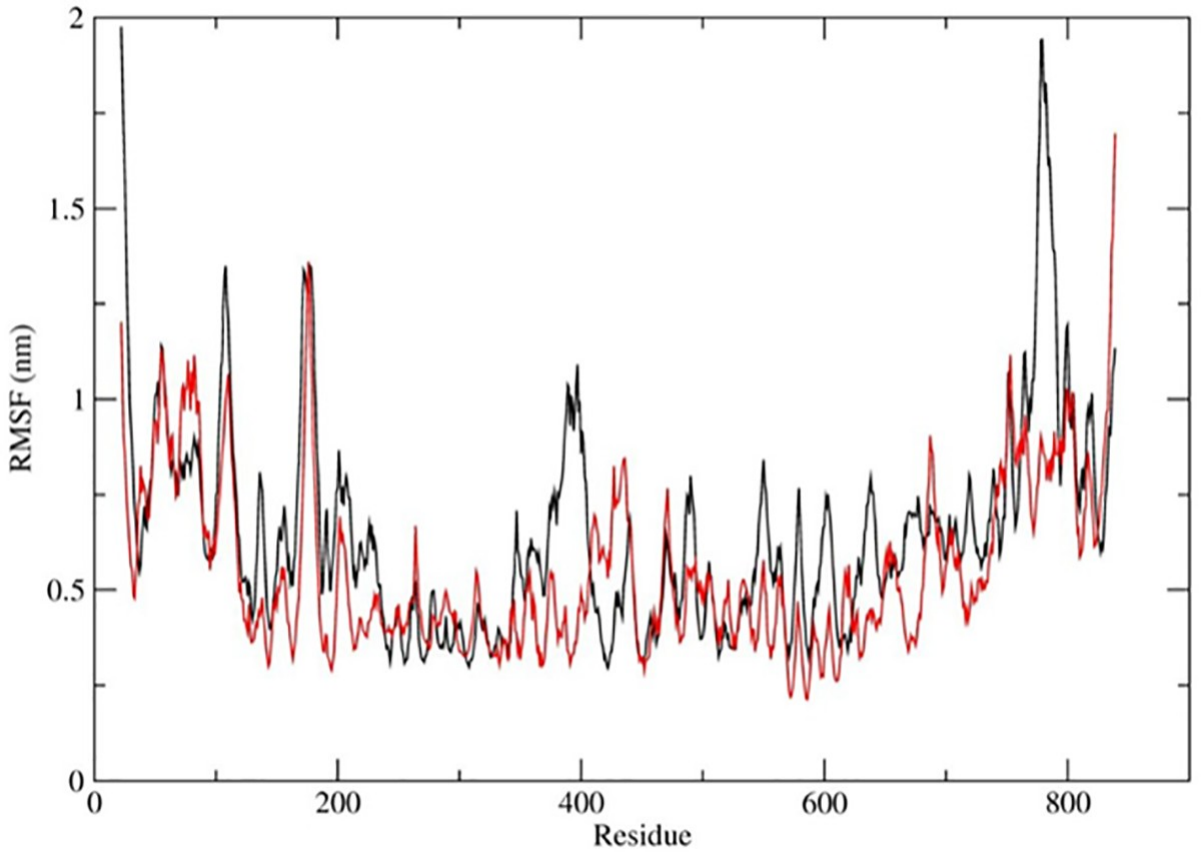
Analysis of simulation trajectory of native and mutant LEPR at 300K. RMSF of the Cα atoms. The color scheme is as follows: native: black and mutant: red.

Fig 1 presents the RMSF graph of the native and mutant LEPR and shows differences in flexibility between the native and mutant protein. It clearly shows that the native and mutant leptin structure at initial residues (between 1–310) exhibited comparable fluctuation which was between ~0.45 mm to ~1.2 mm (Fig 1). Amino acid residues between 1 to 75, 260 to 310 and 405 to 430 in the mutant LEPR show more flexibility than the native protein. But after amino acid number 310 till the end, the mutant exhibited more rigidity as compared to the native protein, and the average fluctuation was ~0.65 mm. On the other hand, native protein showed more flexibility when compared to the mutant and the average fluctuation observed was ~0.91 mm (Fig 1).

**Fig 2:**
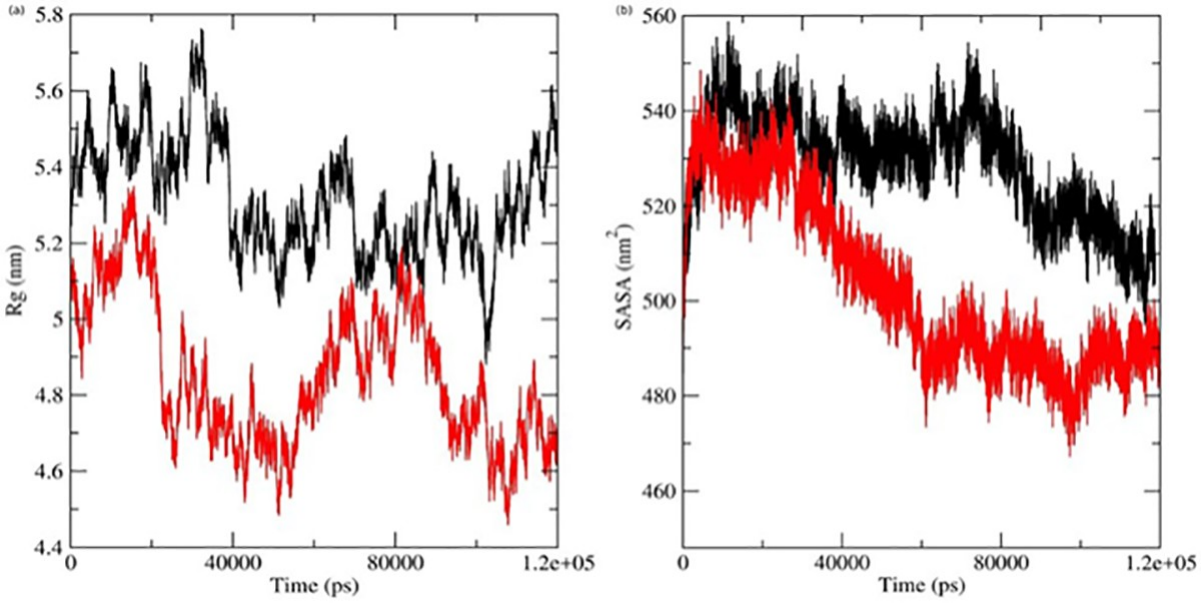
Time evolution of trajectories shown as a function of time at 300 K. (a) Rg of the backbone Cα atoms (b) Solvent-accessible surface area (SASA). The color scheme is as follows: native: black and mutant: red.

The Rg plot of the alpha-carbon atoms of the native and mutant proteins versus time at 300 K was obtained, and the results are presented in Fig 2A. The mutant structure shows a dramatic reduction in Rg values from starting of the simulation till 9 ns, while the native protein exhibited stable and expanded conformation when compared to the mutant. After 9 ns until 12 ns, native LEPR shows Rg deviation between ~5.6 mm to ~5.4 and maintained its expanded conformation, while mutant exhibited Rg deviation between ~5.1 mm to ~5.4 mm and attained compact conformation. Between the time periods of 12 ns to 40 ns, native protein fluctuate between Rg values of 5.3 to 5.7 nm, while mutant protein showed fluctuation between 5.29 to 4.46 nm. After 40 ns till end of the simulation, native protein maintain the average Rg value of 5.2 nm while in mutant this duration values varied much between 4.5 to 5.2 nm.

The overall conformational changes were further validated by the SASA graph which was plotted against the simulation time (Fig 2B). In the mutant LEPR structure SASA values were between ~540 mm2 to ~470 mm2, while the native LEPR had values between ~555 mm2 to ~500 mm2. In this graph also mutant LEPR exhibited sudden fall in SASA values till 18 ns and were stable afterward until the end of the simulation, while native LEPR structure showed stable SASA value between 8 ns to 75 ns and did not show an immediate fall.

**Fig 3:**
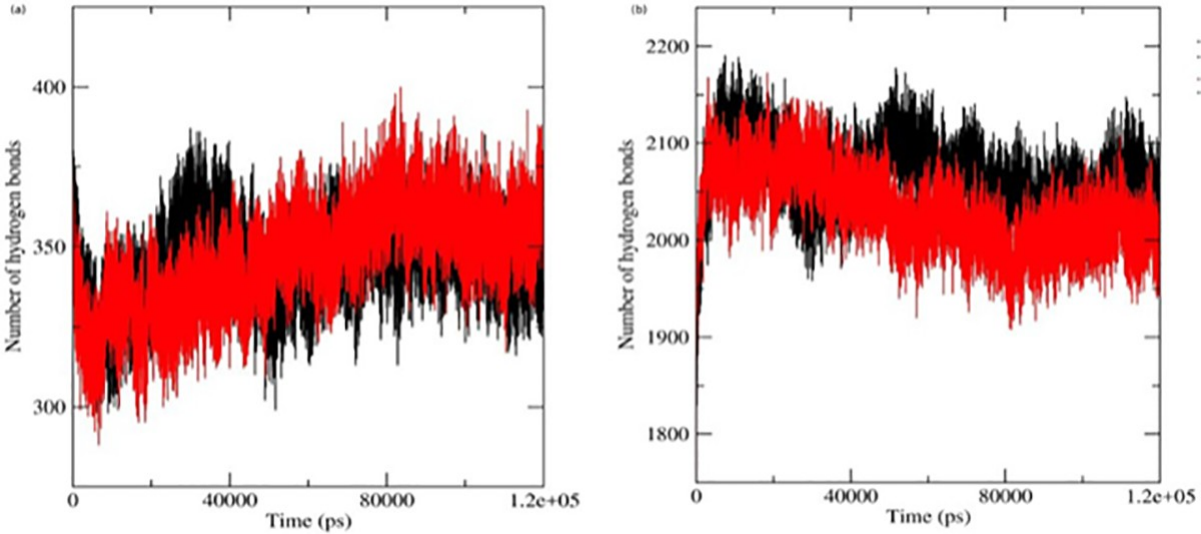
Time evolution of hydrogen bonds in simulation trajectory of native and mutant leptin at 300K. (a) Average number of protein-protein intra hydrogen bonds in leptin protein (b) Average number of protein-solvent inter hydrogen bonds in leptin protein. The color scheme is as follows: native: black and mutant: red.

Intra-molecular (inside the protein molecule with its nearby residues) and inter-molecular (between protein-solvent) hydrogen bond analysis were carried out to observe the bond formation in native and mutant LEPR structures. The H-bond pattern presented a precise idea of molecular intercommunication and confirmed the change in atomic plasticity and geometrical arrangement of a protein molecule in their three-dimensional space. In this analysis, mutant structure showed ~360 intra-molecular h-bonds and ~2030 inter-molecular h-bonds (Fig 3A). On the contrary native structure showed ~339 intra-molecular h-bonds and ~2100 inter- molecular h-bonds (Fig 3B).

**Fig 4:**
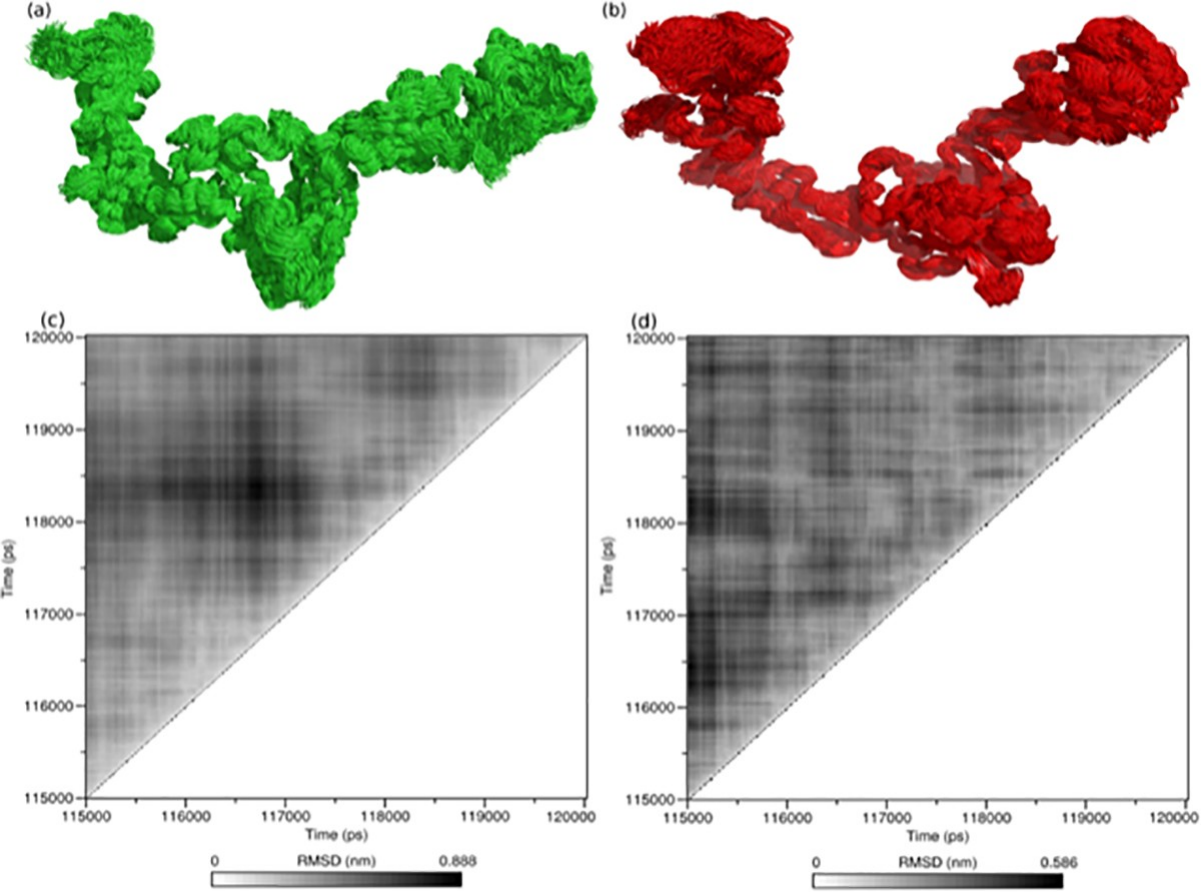
Superimposition of the different conformers and cluster index of native and mutant leptin receptor in MD simulation. (a) native conformations and (b) mutant conformation, cluster index of (c) native and (d) mutant. The color scheme is as follows: native: green, mutant: red, mutation position residue Gln 223 and Arg 223; magenta.

The sampled configurations in each trajectory were clustered applying Daura’s method [50]. Fig 4 presents the results of how the Gln223Arg substitution affects the flexibility of the local region.

To observe the atomic movement at structural front and to better describe the conformational transition during simulation, we clustered the sampled configurations in each trajectory on the basis of the Daura's method [50]. Cluster analysis was observed between the time periods of 115 ns to 120ns. In this analysis, native formed 286 clusters with average RMSD of 0.4261 while mutant structure showed only 225 clusters with the average RMSD of 0.3020. More number of clusters and RMAS values showed that the conformation is highly flexible in the 3D space. We also plotted the superimposition of the different conformers and RMSD distribution of native and mutant LEPR during cluster analysis in MD simulation. These are depicted in Fig 4A–4C.

## Discussion

In this study, we have attempted to relate a substitution in LEPR with alterations in the structure of the receptor and their association with anthropometric, biochemical and hormonal variations in a group of normal weight and obese Saudi women. This polymorphism, an A to G transition in the gene, results in the substitution of a neutral amino acid, glutamine, by a basic amino acid, arginine at position 223 (Gln223Arg). We identified all three genotypes AA, AG and GG in the Saudi females and the frequencies of the A and G alleles were compared with those reported for other populations in NCBI databases [22]. The wild-type A occurs at the highest frequency in the Saudis (0.798) and the results are closer to the frequencies reported for the British (0.610), Puerto Ricans (0.610) and Colombians (0.596), followed by the Punjabi in Pakistan (0.573), Luhya in Kenya (0.520), Toscani in Italy (0.561), Europeans (0.531) and Americans (0.563). The East Asian including Chinese (0.131) and Japanese (0.154) have the lowest frequency of ‘A’ allele. These wide variations in allele frequencies in different populations may be due to multiple contributing environmental and genetic factors.

The relationship between Gln223Arg and obesity has been investigated earlier and recently, Mahmoudi and coworkers have shown the association with obesity in Iranians [25] and Paracchini and coworkers [26] have reported the results of "HuGE” study, showing an association. On the other hand, in a meta-analysis reported recently [27], no association between Gln223Arg in LEPR and obesity was observed. These contradictory results may be explained on the assumption that populations behave differently due to the presence of other genetic variation which influences the association with a disease either positively or negatively or neutralizes the effect of a damaging SNP. During this study, the genotype and allele frequencies in the obese and the normal weight women were compared and no significant difference were seen in the frequency of the A and G alleles. The genotype frequencies were different, but significance was shown only in the AA vs AG frequencies. Interestingly, the results of this study point to the possible involvement of Gln233Arg in inducing changes in obesity related parameters.

In the obese women compared to the normal weight women, all studied parameters except ghrelin and HDL-C, were significantly higher, clearly pointing to the involvement of obesity in altering lipid and hormonal parameters. However, when we separated this group on the basis of the LEPR genotypes, several differences were observed within both lean and obese groups. Firstly, the BMI and waist and hip circumference were significantly higher in the GG homozygotes and AG heterozygotes, compared to the AA homozygotes both in the normal weight and the obese individuals (Table 3). Thus, indicating an association between the mutant genotypes (AG and GG) and weight gain. Lipid levels were not significantly different in the three genotypes, except cholesterol and LDL-C were elevated in AG genotype in the obese women. Hence, our results show that the presence of the G allele increases the risk of anthropometric and lipid abnormalities, particularly in the obese group. Other studies have also reported variations of lipid parameters in different LEPR genotypes. Wu et al. [51] reported in a Chinese population that the AA and AG genotypes associate with lipid abnormalities, where HDL-C levels were significantly lower. Domínguez-Reyes et al. [52] showed that in the presence of the AA and AG genotypes of Gln223Arg, the dietary fat intake modifies the risk of hyperlipidaemias in young subjects. Our results show that the presence of the G allele increases the risk of anthropometric and lipid abnormalities, particularly in the obese group.

Furthermore, variations in the level of hormones were also seen in the different LEPR genotypes where ghrelin showed the maximum abnormality as it was significantly lower in the GG genotype compared to the AA and AG genotypes, both in the normal weight and obese women., Ghrelin plays an major role in metabolism and appetite regulation and may participate in the energy balance during sleep. Wu et al. [51] showed that ghrelin associated negatively with body weight, waist circumference, BMI, body fat, serum leptin level and positively with HDL-C. Our results verify these findings, where in the AA genotype the ghrelin and HDL-cholesterol are higher, while, BMI, W/H ratio and leptin levels are lower. The reverse is true in the individuals with GG genotype. In the obese females, leptin and insulin both had the lowest level in the AA genotype and the highest in the GG genotype and the variations in the results of AA vs. GG and AA vs AG were significantly different. The GG seems to predispose to insulin resistance, and this was confirmed in our study as the HOMA-IR is significantly higher in the GG genotype in the obese women. HOMA-IR is a criterion for assessing β-cell function and insulin resistance from basal (fasting) glucose and insulin or C-peptide concentrations. Our results showed a strong association between genotype GG with insulin resistance. In contrast to our findings, in a study on Brazilian children, Queiroz et al. [53] reported that the A allele of Gln223Arg conferred a higher risk for altered insulin, and HOMA-IR in overweight adolescents. They suggested an increased risk for cardiovascular diseases and/or type 2 diabetes, later in life for individuals carrying the 'A' allele. This finding contradicts the results of the present study, where we have shown that allele ‘A’ occurs at a high frequency in Saudis, though it does not associate with higher glucose level or insulin resistance.

Leptin levels were significantly higher in the obese women with GG genotype compared to the AA genotype (p<0.0001). Normally, leptin is considered as an important hormone in reducing obesity, but extensive studies have revealed that obese individuals have a higher leptin level, and this is shown as a result of ‘leptin resistance’. “Leptin resistance” is defined as the failure of exogenous or endogenous leptin to promote the desired neural, behavioral and metabolic alterations. It is well documented that the hormone levels are elevated if there is a receptor or post receptor defect in hormone action. The defects in binding of leptin to the receptor or some post receptor defect may result in excessive secretion of the hormone, raising its level significantly. It is possible that the women with GG genotype of Gln223Arg suffer from a leptin resistant state and hence, have elevated leptin levels. The assessment of leptin resistance is difficult as it is a state of poor responsiveness to leptin. Myers and coworkers have stated: “*A pragmatic approach to leptin resistance and therapeutic leptin action focuses not on defining clinical leptin resistance in a universal manner*, *but rather on assessing leptin sensitivity*: *Which individuals are likely to respond to leptin and/or can be sensitized to exogenous leptin*?”[54]. Earlier studies have shown that the mutation Gln223Arg does not confer a defect in the binding between leptin and LEPR, pointing to post receptor defect leading to elevated leptin.

A strong association has been reported between leptin and insulin resistance in the obese group [55]. This was confirmed during our study, as we observed a correlation coefficient (r) of 0.684 (p = 0.0001) in the obese group, compared to r = -0.054, p = 0.684, in the normal weight group. Interestingly, both leptin and HOMA-IR were significantly higher in the GG genotype of LEPR compared to the AA genotype. Indicating role played by Gln223Arg in LEPR in influencing the insulin resistance in obese group of Saudi women. Several factors have been proposed to explain the mechanisms involved in the development of insulin resistance and include obesity, inflammation, hyperlipidemias and others. We can propose that one of the factors could be Gln223Arg polymorphism in LEPR gene. However, detailed studies are necessary to confirm this proposal.

In order to rationalize the deleterious effect of leptin mutation which was screened in our *in vitro* experiments, we conducted atomic molecular dynamics simulation of native and mutant forms of leptin receptor. As is well documented, the structural conformation of a protein molecule defines its cellular function and changes in conformational behaviour due to mutation would lead to malfunction. The MD studies revealed several interesting aspects of the LEPR structure in the native and mutant protein, Our MDS trajectories analysis of native and mutant LEPR protein showed that, due to Gln substitution at the 223rd position by Arg, the receptor acquired a rigid conformation in its 3D space. The rigidity in the mutant and flexibility in the native structure were clearly indicated in RMSF analysis. This rigidity could be the reason leading to its malfunctioning. RMSF graph clearly showed that native and mutant LEPR structure at the initial residues (between 1–310) exhibited fluctuation ranging between ~0.45 mm to ~0.75 mm (Fig 1), but not in the later regions. We further organized Rg and SASA analysis to confirm the altered conformational behavior of LEPR protein due to the mutation. Rg analysis revealed the compactness and packing arrangement of amino acid in 3d space with time. The mass-weighted RMS distance of a cluster of atoms from their center of mass, showed expanded conformation when compared to the mutant. And this may be due to N-terminal residual motion which was observed in the RMSF graph. Overall, the native protein showed a compact conformation when compared to its mutant. This might be due to the N-terminal residual motion which was observed in RMSF graph.

The SASA graph indicated overall conformational changes which were plotted during the simulation process. These observations well corroborated with Rg and confirmed the rigid nature of mutant LEPR protein. In the Rg graph also the mutant exhibited sudden fall in SASA values till 18 ns and was stable afterwards until the end of the simulation, while native structure showed stable SASA value between 8 ns to 75 ns and did not show an immediate fall.

These observations were well corroborated with Rg and confirmed the rigid nature of mutant leptin protein. Alteration in the rigid and flexible structure pointed to a dynamic nature and changes in the bond formation of the structure during simulation. The numbers obtained clearly indicated that the mutant has a higher tendency to form intra- molecular h-bonds and less inclination to form inter-molecular h-bonds when compared to the native structure. The rigid and compact nature of the mutant compared to the native structure, was further validated by its bond formation nature.

To observe the atomic movement at the structural front and to better describe the conformational transition during simulation, our computation analysis explained the deleterious role of Gln223Arg in LEPR at the atomic level. It was clear that the mutation (Gln223Arg) not only affects the flexibility at local region but it has a global impact on the structure. This finding will help to understand the pathology and will pave the path toward drug formulation research.

From the results of this study, we propose that the Gln223Arg polymorphism affects the structural flexibility of the LEPR protein. The binding of leptin to the receptor LEPR, may not be affected by this change. However, it is possible that the conformational changes that occur in the LEPR upon leptin binding, which is essential for the activation of the JAK2, which in turn phosphorylates intracellular tyrosine residues, may not take place normally, hence leading to leptin resistance and diminished action. Our results point to a leptin resistance in the individuals with the mutant allele. Further structural studies are required for confirmation of this proposed mechanism.

Finally, this investigation was conducted only on females and this was one of the main limitations of this study. Since LEPR gene is an autosomal gene, it is expected that the frequency of the A and G alleles in the males will not be very different. The molecular dynamic study results are also not expected to be influences by the gender. However, further more detailed studies are warranted to compare the results of genotype and allele frequencies in LEPR gene in males and females.

## Supporting information

S1 Data The demographic, metabolic and leptin receptor gene polymorphism (rs1137101) data from normal weight and obese Saudi women. (SAV)
